# Retraction Note to: Inhibition of Tomato Yellow Leaf Curl Virus (TYLCV) using whey proteins

**DOI:** 10.1186/s12985-023-02100-4

**Published:** 2023-06-19

**Authors:** Ashraf M Abdelbacki, Soad H Taha, Mahmoud Z Sitohy, Abdelgawad I Abou Dawood, Mahmoud M Abd-El Hamid, Adel A Rezk

**Affiliations:** 1 Plant Pathology Department, Faculty of Agriculture, Cairo University, Giza, 12613 Egypt; 2 Dairy Science Department, Faculty of Agriculture, Cairo University, Giza, 12613 Egypt; 3 Biochemistry Department, Faculty of Agriculture, Zagazig University, Zagazig, Egypt; 4 Virus and Mycoplasma Department, Agriculture Research Center, Giza, 12619 Egypt

**Retraction to: Virology Journal 2010, 7:26**

**http://www.virologyj.com/content/7/1/26**

The Editor in Chief has retracted this article because of multiple concerns about image overlaps, namely:

- Rows A1 and B1 of Fig. 2 appear similar;
- The three last blots in line B4, Fig. 1 and line B4, Fig. 2 appear to be repeating;
- The first two blots in lines A2 and A3. Fig, 2, appear to be repeating;
- Rows B1 and B6 in Fig. 3 appear similar.

The authors did not respond to a request for clarification nor to correspondence from the Editor about this retraction.

